# Smart toilets for monitoring COVID-19 surges: passive diagnostics and public health

**DOI:** 10.1038/s41746-022-00582-0

**Published:** 2022-03-30

**Authors:** T. Jessie Ge, Carmel T. Chan, Brian J. Lee, Joseph C. Liao, Seung-min Park

**Affiliations:** 1 Department of Urology, Stanford University School of Medicine, Stanford, CA USA; 2 Department of Radiology, Stanford University School of Medicine, Stanford, CA USA; 3 Molecular Imaging Program at Stanford, Stanford University School of Medicine, Stanford, CA USA; 4 Department of Mechanical Engineering, Sungkyunkwan University, Suwon, Republic of Korea

**Keywords:** Diagnostic markers, Gastrointestinal diseases, Diagnosis, Translational research

## Abstract

Continued COVID-19 surges have highlighted the need for widespread testing in addition to vaccination for disease containment. SARS-COV-2 RNA can be found in faecal matter, making human stool another potential source for COVID-19 diagnostics. In this commentary, we highlight potential strategies to use a smart toilet platform to passively monitor COVID-19 surges, enabling earlier detection of infected individuals and promoting public health.

## Introduction: Passive monitoring for COVID-19

The coronavirus disease 2019 (COVID-19) pandemic has been managed with social distancing and masking, testing and isolation, and vaccination. As new variants of severe acute respiratory syndrome coronavirus 2 (SARS-CoV-2) continue to emerge, there is a general consensus that the COVID-19 pandemic will transition to an endemic status^1^. This may necessitate continued widespread and frequent testing to detect and control new outbreaks. Standard COVID-19 testing is currently performed with nasopharyngeal or nasal mid-turbinate swab tests. Though the latter has facilitated self-testing and diminished some of the discomfort associated with testing, these methods still require the individual to physically travel to a testing site or to drop off their sample. As the pandemic persists, psychological fatigue may lead to people becoming less sensitive to COVID-19 control^2,3^, decreasing personal incentives for COVID-19 testing and thereby making passive monitoring systems attractive for public health.

## SARS-CoV-2 RNA can be found in human stool

Gastrointestinal symptoms are among the primary manifestations of COVID-19, contrary to the common perception of the SARS-CoV-2 infection as a respiratory illness^4^. A number of studies have described the gastrointestinal tropism of SARS-CoV-2^5^. SARS-CoV-2 can be found in the faeces even after respiratory symptoms resolve and nearly 5 weeks after nasopharyngeal viral RNA tests become negative^6^, implying potential (but controversial) faecal–oral transmission^7,8^ and the possibility of longitudinal faecal testing as a surveillance tool. While China has implemented anal swabs for COVID-19 testing in select cases^9^, faecal testing for COVID-19, whether through swabs or individual stool sampling, has not become routine due to its intrusive and unpleasant nature.

Another manner of faecal SARS-CoV-2 examination uses wastewater to investigate the cumulative viral material shed from communities. Measuring SARS-CoV-2 RNA concentrations in wastewater may enable the prediction of possible adjacent viral hot-spots, and sequencing it can identify emerging variants or establish viral mapping of the most transmissible variants. A recent study^10^ showed strong correlations between the sludge SARS-CoV-2 virus RNA concentration curve and COVID-19 epidemiology curves (e.g. test results, hospital admissions), suggesting the concentration of viral RNA in wastewater would be an indicator to monitor the extent of the virus. However, variability in the wastewater systems (e.g. presence of industrial waste and/or runoff, differences between composition wastewater in various communities) serves as a confounding factor^11^. Additionally, direct conversion of viral RNA concentrations in wastewater to the prevalence of the SARS-CoV-2 virus in a community is not possible as no normalisation of the viral concentration per person has been determined^11^. Similarly, there is a lack of data surrounding the interpersonal variability in viral RNA excretion over the course of COVID-19 disease. As a result, wastewater analysis lacks precision for more granular estimations of COVID-19 prevalence. A similarly passive but more individualised approach to faecal viral RNA surveillance will improve access to COVID-19 testing and limit the spread of infection. Current methods of individual stool sampling require the subject to actively handle their own stool to obtain the specimen which limits its widespread use. Recently emerging “smart toilet” technologies represent an opportunity to fully automate stool collection and faecal biomarker detection.

## Smart toilet platforms for COVID-19 screening/diagnostics

**Fig. 1:**
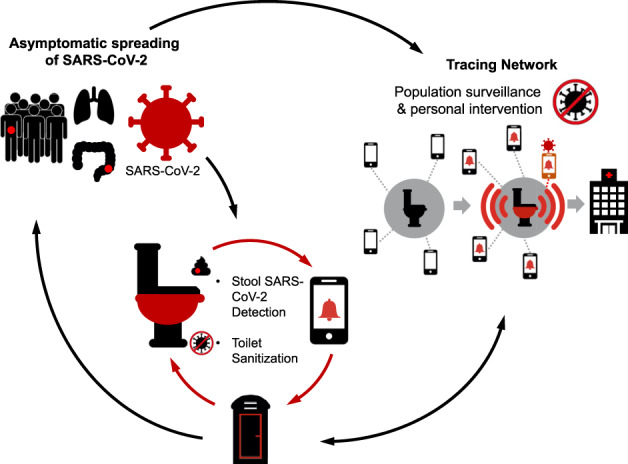
The COV-ID (Coronavirus: Integrated Diagnostic) toilet network for surveillance of COVID-19. The COV-ID toilet will serve as a centralised diagnostic centre in essential businesses to detect COVID-19 infection amongst asymptomatic population and to facilitate early diagnosis and isolation. Stool sampling will be automated with quick turn-around time for viral RNA testing, and the toilet seat/surfaces will be sanitised in between users. The user will register with the health portal or a tracing network (can be performed on a mobile device while on the toilet), which will then alert them to their test results (ideally within 15 min), link them with the medical care, provide advice and guidelines from physicians for isolation. Aggregate data can also be used for population surveillance to assess community burden.

As shown in Fig. 1, a public health strategy is proposed for infectious disease surveillance using toilets fitted with a stool collection and analysis system that performs fully automated and passive diagnostics. A smart toilet platform, namely “Coronavirus: Integrated Diagnostic (COV-ID) toilet” would consist of a mountable bidet-style attachment equipped with modules for automated faecal sample collection and processing, faecal RNA isolation and detection with in situ ultrafast nucleic acid amplification tests (NAAT), and effective sanitisation methods. COV-ID toilets can be installed in highly trafficked, public areas ranging from shopping malls and sporting arenas to schools and hospitals. While a person sits down to use the toilet, they can scan a Quick Response (QR) code to consent to COVID-19 stool testing. If permitted, the COV-ID toilet will detect defecation events and automatically sample and test the stool for COVID-19. Test results are reported in minutes—to the individual’s smartphone, if desired, and to an anonymized tracing system. The individual with a positive result will be provided with information to determine quarantine protocols and further confirmatory testing. The tracing system can be linked to the existing Bluetooth^®^ contact tracing systems implemented by Apple^12^ and Google^13^ for COVID-19 exposure notifications. A network of COV-ID toilets could also augment and refine maps of COVID-19 prevalence, and provide more real-time information to guide decisions about travel. The same testing principles can also be applied to other infectious diseases with faecal–oral transmission, such as norovirus or bacteria related to gastroenteritis like Shigella, to help prevent and control outbreaks in real time.

The prerequisites for successful deployment of COV-ID toilets in diverse communities are: (1) A fast turn-around time, ideally within 15 min from sample-to-answer. In this regard, ultrafast NAAT are preferred to shortening the turn-around time of conventional NAAT methods (e.g. PCR) from a couple of hours down to 8–15 min^14–17^. In addition, multiplexed and parallel runs of COVID-19 detection in the system can ensure immediate toilet availability to other users. (2) Full automation from sample acquisition to signal generation must be performed in the background in order not to interfere with normal human behaviour on the toilet. This automation will maintain high user compliance and lower human intervention. (3) To provide a hygienic environment and eliminate cross-contamination between tests, rigorous sanitation/sterilisation (e.g. ultraviolet steriliser) has to be performed on the analytical devices and on the surface of the toilet. (4) As an Internet of Things (IoT) device, the COV-ID toilet should be securely connected to a centralised network, which enables active communication with users and intra-network surveillance. (5) While the user may elect to provide identifying information such as a cell phone number in order to receive their own test results, the results must be de-identified prior to upload to the tracing network in order to maintain the user’s privacy.

Other important biological/clinical data can be obtained by the COV-ID toilet. Body temperature and oxygen saturation, which are known to be critical parameters of COVID-19 infection, can be obtained by integrating temperature and photoplethysmography sensors into the toilet seat^18^. Stool morphology analysis can identify diarrhoea, which is a potential symptom of COVID-19. This feature can be easily adopted from a previous smart toilet study with deep learning and computer vision^19,20^. By collecting other supplementary data (temperature, oxygen saturation, and stool morphology) in tandem with COVID-19 stool testing, the COV-ID toilet may enable comprehensive profiling of COVID-19 infection.

## Contributions to public health and research

To date, there have been few studies on if and how the presence of SARS-CoV-2 viral RNA in the stool correlates with infection time-course, severity, or symptomatology. COV-ID toilet networks can provide data on a public health scale, but individual units can also be provided to research study participants to easily generate individualised, longitudinal data. As previously mentioned, the lack of data surrounding the variability in viral RNA faecal excretion over the course of COVID-19 disease has limited the utility of wastewater analyses. An automated system can help promote research participation, as current methods require the individual to place a collection device in the toilet and then directly collect and handle their own stool, which some may find odious. The COV-ID toilet platform will help inform the scientific community on the behaviour of SARS-CoV-2 in the gastrointestinal system, including viral persistence in stool and potential faecal–oral transmission, and the optimal frequency of testing, as well as provide complementary information such as temperature, oxygen saturation, and presence of diarrhoea. It will provide an alternative form of testing to complement the nasopharyngeal swab—one that is non-invasive, more amenable to serial testing, and able to be performed passively in the background without exposing the testers to the virus.

Once a significant number of COV-ID toilets are deployed in public, the COV-ID toilet will augment COVID-19 testing capabilities to provide a better sense of the true infection rate (or prevalence) of an area. It will also allow for earlier detection of infected individuals which could herald an outbreak by continually testing an entire community—including those who are potentially asymptomatic. Long-term, one can compile the data from COV-ID toilets in multiple locations to help predict and prevent clusters of outbreaks. An uptick in positive test results in a single facility may indicate that that location needs to be closed and disinfected, individuals living in that community should be alerted to be tested, and newly infected individuals should be isolated. Meanwhile, increases in multiple locations within highly organised settings may help guide decisions on shelter-in-place policies.

## Barriers to passive monitoring in the pandemic

However, the success of such a strategy will hinge on user acceptance. Certain settings such as military barracks or naval ships, which have experienced rapid and widespread outbreaks^21^, are virtually guaranteed to have their inhabitants use the COV-ID toilet if it is installed, but consent is likely required for individualised testing. In the general public, people may avoid the toilets or not consent to testing if they feel there is a risk to personal privacy or are otherwise averse to testing. On the other hand, motivated individuals may find the COV-ID toilets a convenience and lower barrier to rapid, personalised testing. The establishments themselves would also have to be incentivized to install the COV-ID toilets, likely with tax credits or some other measure. Considering the enormous adverse economic impact of COVID-19, private and public investment into installing the COV-ID toilet will likely be justified. In addition, COV-ID toilets can be designed in bidet-style attachments that are easily installed without replacing existing infrastructure, which would significantly alleviate economic burden on installing such devices. Interestingly, our survey in the previous publication^19^ indicated that smart home users are more acceptable toward smart toilets, implying smart city residents may have a great interest in such technologies. Smart cities are municipalities that are equipped with connected technologies and IoT solutions to enhance many aspects of human life from critical infrastructure and public safety and health. These opportunities not only resolve user acceptance issues but also alleviate economic burden of installing such devices. Thus, it is a potential solution that smart toilets (including COV-ID toilets) can be employed as a healthcare sentinels in the city when planning and a pilot clinical study can be conducted for clinical validation.

One can hypothetically envision a scenario where COV-ID toilet test results are anonymized by default and only submitted to a tracing network for public health interventions. In this context, would individual consent be required for a non-invasive, automated test? There are no other similar strategies currently in use which can be modelled upon, and strategies such as wastewater sampling are not directly comparable as the COV-ID toilet is individual-facing. The novelty of non-invasive, passive diagnostics in public health will require serious considerations on the ethics and privacy front.

The COV-ID toilet platform will be useful not only for the current COVID-19 pandemic, but can provide a robust infrastructure for monitoring future novel disease, especially as epidemics become increasingly frequent in the setting of urbanisation and globalisation^22^. This century has already seen outbreaks of severe acute respiratory syndrome (SARS, 2002), swine flu (2009), middle east respiratory syndrome (MERS, 2012), Ebola (2013), Zika (2015), and Dengue fever (2016). The COV-ID toilet platform can also be used to track and prevent local outbreaks of diseases like gastroenteritis by testing for causative agents such as norovirus or Salmonella.

## Conclusion

We envision passive toilet-based sampling and testing as a key strategy in advancing precision health-based diagnostics^23–25^ by tapping into the wealth of information contained in urine and stool, which has been too frequently flushed away. A smart toilet can access this underutilized data without requiring significant user intervention, and even circumvent potential behavioural fatigue associated with routine COVID-19 testing. While more research is needed regarding the relationship between faecal viral shedding, faecal–oral transmission, and overall infectivity, toilet-based sampling can improve the accessibility of COVID-19 testing and provide more granular data on infection rates and outbreaks as shown in Fig. 1.

### Reporting summary

Further information on research design is available in the Nature Research Reporting Summary linked to this article.

## Supplementary information

Reporting summary (Updated 2/5/2022)
